# Auxin-Mediated Lateral Root Development in Root Galls of Cucumber under *Meloidogyne incognita* Stress

**DOI:** 10.3390/plants13192679

**Published:** 2024-09-24

**Authors:** Baoling Ren, Xin Guo, Jingjing Liu, Guifang Feng, Xiaodong Hao, Xu Zhang, Zhiqun Chen

**Affiliations:** 1 College of Life Sciences, Linyi University, Linyi 276000, China; r33088286821@outlook.com (B.R.); 18369422404@163.com (X.G.);; 2 School of Resources and Environment, Linyi University, Linyi 276000, China

**Keywords:** cucumber, auxin, *Meloidogyne incognita*, lateral root, root galls

## Abstract

Root-knot nematodes induce the formation of feeding sites within the host roots and the relocation of auxin into galls results in abnormal lateral root growth. Here, we analyzed the changes in cucumber root architecture under *Meloidogyne incognita* stress and the distribution of auxin in these morphological and molecular root changes. The number of root tips significantly decreased, and regression analysis showed a positive relationship between the size of root galls and the numbers of nematodes in galls compared with the lateral roots on galls, emphasizing the effect of nematode parasitism on root development. Data generated via a promoter-reporter system using the transgenic hairy root system first characterized the auxin distribution during nematode parasitism in cucumber. Using *DR5:GUS* staining of root galls, we further detected the expression of *CsPIN1* and *CsAUX1*, which regulate polar auxin transport. The results showed that both *CsPIN1* and *CsAUX1* were induced in galls, and the relative expression of the two genes significantly increased at 21 DAI. The TIBA treatment, which can disrupt polar auxin transport inhibited the numbers of cucumber root tips and total length following increasing concentration gradients. Moreover, the numbers of galls were significantly affected by TIBA treatment, which showed the vital role of auxin during nematode parasitism. Our findings suggest that the transportation of auxin plays an important role during gall formation and induces cucumber lateral root development within nematode feeding sites.

## 1. Introduction

Root-knot nematodes (*Meloidogyne* spp.) are the major nematode parasites in vegetable cultivation. As sedentary nematodes, they establish an intimate relationship with the host roots and cause devasting damage by destroying diverse root functions. Second-stage juveniles (J2) of the root-knot nematodes (RKNs) infect the root at the root tip area and move intercellularly to find the feeding sites [1]. RKNs select several cells, usually located in the pro-vascular area, to establish a feeding site upon injection of stylet secretions [2,3], and these cells then become giant cells (GCs) to supply nutrients. The resulting giant cells may contain hundreds of polyploid nuclei, and characteristic galling of the surrounding tissue is induced by the enlarged and divided surrounding pericycle and cortical cells. Previous studies showed the parallel molecular pattern between lateral root (LR) development and RKN gall formation due to a similar regulatory gene pathway [4]. Moreover, different tagged lines in LRs and galls demonstrated the same response pattern [5] which suggests a relationship between LR emergence and RKN feeding site formation.

Plant hormones are known to regulate plant growth and development. Auxin has long been suspected to play a crucial role in nematode infection, and reporter studies have shown the presence of auxin during feeding site formation [6]. Due to the vital role of auxin in nearly all plant growth and developmental processes [7], especially the regulation of cell division and root primordia inducement [8], our knowledge of its role during nematode parasitism still needs to be explored. Studies using the promoter-reporter system demonstrated the specific transportation and distribution of auxin in the initial feeding cells induced by RKN in *Arabidopsis thaliana* [9] and the activation of auxin-response *DR5:GUS* reporter in the neighboring cells around GCs [6].

During plant root growth, auxin also regulates the initiation, emergence, and elongation involved in lateral root development [10]. Mutants with a disrupted auxin signaling cascade or with a defect in the auxin transport system disturbed the lateral root phenotype [11,12]. The role of auxin transport governed by AUX1/LAX and PIN proteins was studied during GC development upon RKN infection [13]. The AUX1 protein is plasma membrane-localized and has been shown to actively transport auxin into cells. The aux1 mutants had defects in auxin movement from the root apex to the distal elongation zone [14] and showed significantly fewer and smaller galls [13]. For lateral root development, *AUX1* appears essential for efficient auxin uptake by expanding epidermal cells [14]. A marked decrease in the numbers of lateral roots is observed when the expression of *AUX1* is silenced [15,16]. There is less information reported on the role of PIN proteins in root-nematode interactions. PINs are essential for normal organogenesis and/or auxin-dependent tropic responses [17]. *AtPIN1* has been shown to accumulate in the vascular tissue of roots [18] and is also localized in the endodermis, which is the supposed gravity-sensing tissue in the transition zone of cucumber seedlings [19]. The formation of nematode feeding sites in roots was similar to lateral root development, which is induced by auxins through the regulation of PIN proteins [12].

Cucumber (*Cucumis sativus* L.), a worldwide cultivated vegetable crop, suffers great damage from root-knot nematode disease, especially *Meloidogyne incognita*. In greenhouse cucumber cultivation affected by root-knot nematode disease, we noticed abnormal lateral root growth on root galls. It has been suggested that auxins play a role in the formation of secondary roots that are generated spontaneously due to an infection of *M. incognita* and other RKNs and CNs [20,21]. However, there are still gaps in the knowledge of whether auxins are involved in nematode infection of cucumber roots.

Since many processes in lateral root development and nematode feeding site formation are governed by auxin, the question arises of whether auxin responses are involved in cucumber root gall development and whether they affect lateral root growth in galls. Here, we report on the changes in cucumber root architecture induced by nematode infection and the lateral root plasticity during root gall development. To explore the response of auxin location during nematode parasitism, we performed reporter analysis using a DR5 promoter. Furthermore, the responses of auxin efflux carrier *CsPIN1* and influx carrier *CsAUX1* were analyzed during nematode parasitism. Our study reveals that auxin diffusion induced by nematode infection can be recruited as a regulatory mechanism to coordinate lateral root proliferation in cucumber root galls.

## 2. Materials and Methods

### 2.1. Plant Material and Nematodes

Cucumber (*Cucumis sativus* L.) cultivar “Zhongnong 26” was used in this study. Seeds were surface-sterilized using 3% sodium hypochlorite and germinated on moistened filter paper in darkness at 28 °C. All cucumber plants were grown under long-day conditions (16-h light, 26 °C/8 h dark, 20 °C cycles) with a light intensity of 12,000 Lux. Maintenance of the *Meloidogyne incognita* population was cultured on cucumber in sterilized soil. The egg masses were collected and sterilized with 0.5% sodium hypochlorite for 3 min and then submerged in sterile water at 25 °C for 3 days. Freshly hatched pre-J2s were collected using a 500-mesh screen and stored at 4 °C before the experiments.

### 2.2. Analysis of Root Architecture and Lateral Root Numbers on the Root Galls

To compare the architecture of cucumber root systems under *M. incognita* stress, a pot experiment was conducted. The soils used were collected from a forest site and air-dried for 7 days at room temperature. The soils were then homogenized and sieved with 2-mm meshes. Moreover, the soil was sterilized by γ-irradiation (at 60 kGy) to ensure that no other uncontrolled infection occurred [22]. Sterilized and germinated cucumber seeds were sown into 7 × 10 cm pots. Two-true leaf cucumber seedlings were inoculated with 300 *M. incognita* J2s. Briefly, a 1 mL aliquot of a suspension containing 300 *M. incognita* pre-J2s was used to inoculate into soil around cucumber roots. To prevent root system damage after long-term nematode infection, the cucumber roots were harvested at 14 days after nematode inoculation (DAI) to determine the global change of root architecture. The cucumber root systems were placed on transparent plastic slides and combed carefully apart before being scanned by an Epson expression scanner at a resolution of 300 dpi. Root architecture parameters were analyzed by WinRHIZO software (LC4800-II LA2400; Sainte-Foy, Quebec City, QC, Canada). Moreover, the root galls were sampled at 28 DAI to determine the numbers of lateral roots on the surface in the late stage of nematode development. Photographs of cucumber root galls were further taken, and ImageJ was applied to measure the maximum length of each root gall. At least eight replicates (independent seedlings) were set up for the analysis of root architecture and lateral root numbers.

### 2.3. Promoter Activity Analysis Using Cucumber Transgenic Hairy Roots

The *CsPIN1:GUS* and *CsAUX1:GUS* constructs were generated by fusion of a PCR-amplified fragment (~2000 bp) upstream of the ATG codon and the GUS gene. The primers for vector construction are listed in Supplementary Table S1. The fragments were then ligated to the pCAMBIA1391 vector using a NovoRec^®^ plus One step PCR Cloning Kit (NovoRec, NR005, Shanghai, China). The pCAMBIA1391 vector was equipped with a β-glucuronidase (GUS) reporter gene, which could enable characterization and localization of gene expression. After validation by sequencing, all the constructs were transformed into *Agrobacterium rhizogenes* strain K599 for cucumber hairy root transformation. The auxin-responsive promoter DR5 reporter system is functional to monitor auxin response in different plant tissues. Therefore, the binary construct harboring *DR5:GUS* was also transformed into *Agrobacterium rhizogenes*. The induction of cucumber transgenic hairy roots was performed using strain K599 following the method described previously [23]. Briefly, the collected K599 suspension carrying specific GUS expression vectors was injected into the cotyledonary node. When enough hairy roots were induced at the injection sites, the original root was cut off, and the cucumber plant was supported by the hairy roots in sterilized soil.

To analyze the promoter activity during *Meloidogyne incognita* parasitism, 300 J2s were inoculated into the transgenic hairy roots transformed with the GUS reporter vector. At least 10 independent hairy root systems were analyzed for GUS reporter activity.

### 2.4. Relative Expression of CsPIN1 and CsAUX1

To more intuitively reflect the difference in the relative expression levels of *CsPIN1* and *CsAUX1* in cucumber roots, we divided the infected cucumber root into different parts at different developmental stages. The mock-infected cucumber roots were set as control and the nematode-infected cucumber roots were set up for two parts: Galls (cucumber root galls) and roots (the part of cucumber roots inoculated with nematodes after removing the root galls). The sampling timepoints were set at 3, 7, 14, and 28 DAI.

The sampled root parts at different developmental stages were used for total RNA extraction and cDNA synthesis (Vazyme, Nanjing, China). RNA quantification was performed using a Nanodrop 2000 (Thermo Fisher Scientific, Waltham, MA, USA). The qRT-PCR analysis using SYBR Green Master Mix (Vazyme, Nanjing, China) was performed in an ABI 6500 Real-Time PCR System (Applied Biosystems, Waltham, MA, USA). *CsTUA* (accession number Csa4G000580) was used as an internal control. Relative expression abundance of candidate genes was calculated with the formula 2^−ΔΔCt^. All reactions were performed with four biological replicates. Genomic DNA of hairy roots was isolated using FastPure Plant DNA Isolation Mini Kit (Vazyme, Nanjing, China). All the primers for characterizing *GFP* from hairy roots and qRT-PCR analysis are listed in Table S1.

### 2.5. GUS Staining of the Transgenic Roots

GUS staining of transgenic hairy roots sampled at 3, 7, 14, and 28 days after inoculation with *Meloidogyne incognita* was performed following the protocol of the GUS staining kit (Obiolab, Beijing, China). Observation of histochemical *GUS*-stained root galls was performed by spreading them on a microscope slide and mounting them in chloral hydrate solution (8 g of chloral hydrate, 2 mL of water, 1 mL of glycerol). Moreover, paraffin-embedded sections were used to analyze the tissue localization of *GUS* expression in root galls. For GUS staining observation of galls on paraffin sections, 6 μm paraffin sections were deparaffinized and rehydrated. Representative photographs of cross-sections were taken under a microscope.

### 2.6. TIBA Treatment of Cucumber under M. incognita Stress

To assess the possible involvement of auxin transport in the altered root phenotype, we performed an experiment of exogenous application with 2,3,5-triiodobenzoic acid (TIBA), an auxin polar transport inhibitor. The sterilized and germinated cucumber seeds were sowed into 4 × 7 cm pots filled with sterilized soil. We tested four concentration gradients: 50, 100, 200 and 400 mM. When the first true leaf of cucumber seedlings spread out, 50 mL of different concentrations of TIBA solution was directly watered into the root zone of cucumber seedlings. Water was used as a solvent control. At least eight replicates (independent seedlings) were set up for each TIBA concentration condition. After the application of TIBA, 100 prepared *M. incognita* J2s were inoculated into the cucumber root zone immediately. The cucumber root system was sampled at 14 DAI to analyze the change in root architecture and root galls formed by *M. incognita*.

### 2.7. Statistical Analysis

Statistical analysis was performed using Student’s t-test, and the correlation analysis of the relationship between the numbers of nematodes in root galls and the numbers of lateral roots was performed using linear regression analysis in SPSS 22.0. The confidence intervals were established with a significance of 5% (*p* < 0.05; asterisk). GraphPad Prism 9.0 software was used for graphing.

## 3. Results

### 3.1. Nematode Infection Affects the Lateral Root Development

**Figure 1:**
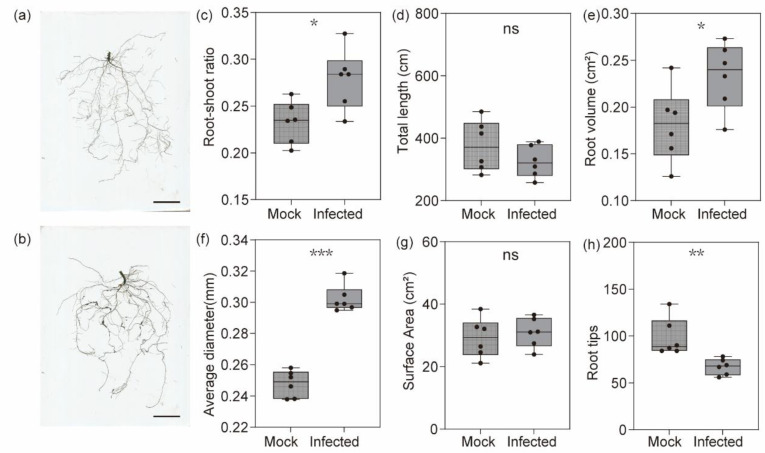
Nematode infection affects cucumber root architecture. (**a**,**b**) Representative pictures of cucumber root system treated with Mock (**a**) and *M. incognita* (**b**). Bar: 5 cm. (**c**) The root-shoot ratio of cucumber treated with Mock and *M. incognita*. (**d**) The total length of cucumber root treated with Mock and *M. incognita*. (**e**) The root volume of cucumber treated with Mock and *M. incognita*. (**f**) The average diameter of cucumber root system treated with Mock and *M. incognita*. (**g**) The surface area of cucumber root system treated with Mock and *M. incognita*. (**h**) The numbers of cucumber root tips treated with Mock and *M. incognita*. Data are presented as the mean ± SE, Student’s *t*-test, n = 6. * *p* < 0.05; ** *p* < 0.01; *** *p* < 0.001; ns, not significant.

Studies have demonstrated that nematode infection can affect plant growth. In this study, the global root architecture of cucumber significantly changed during *M. incognita* parasitism (Figure 1a,b). Nematode infection of the cucumber plant triggered an increased allocation of biomass to the roots (Figure 1c). The root volume and average diameter of cucumber significantly increased after nematode infection, but the total length and surface area did not show obvious differences (Figure 1c–g). The root tips in the whole cucumber root system significantly decreased during nematode parasitism (Figure 1h) which reflected the decreased numbers of lateral roots.

**Figure 2:**
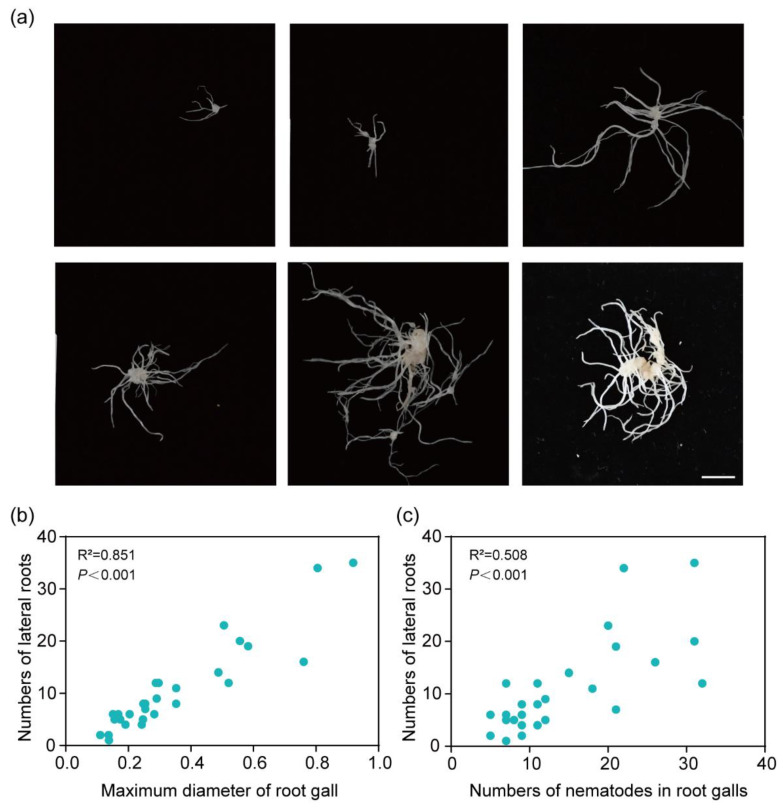
Lateral roots induced in cucumber root galls. (**a**) Representative pictures of cucumber root galls of different sizes and the lateral root growth. Bar: 2 cm. (**b**) The correlation analysis of the relationship between the maximum diameter of root galls and the numbers of lateral roots was performed using linear regression analysis. (**c**) The correlation analysis of the relationship between the numbers of nematodes in root galls and the numbers of lateral roots was performed using linear regression analysis.

Furthermore, the numbers of lateral roots growing on the galls were counted at 28 DAI. Results showed that cucumber roots generated additional lateral roots during nematode parasitism (Figure 2a). Specifically, regressions to show the relationship between the diameter of root galls and lateral roots on galls (Figure 2). As root galls become larger, the lateral roots become more numerous and denser (Figure 2b). Furthermore, we detected the numbers of nematodes in specific galls, which showed that as the number of root-knot nematodes increased, the number of lateral roots on the galls also increased (Figure 2c). These results demonstrated the interaction between lateral root development and nematode parasitism.

### 3.2. DR5 GUS Indicated the Auxin Distribution in Roots and Root Galls

**Figure 3:**
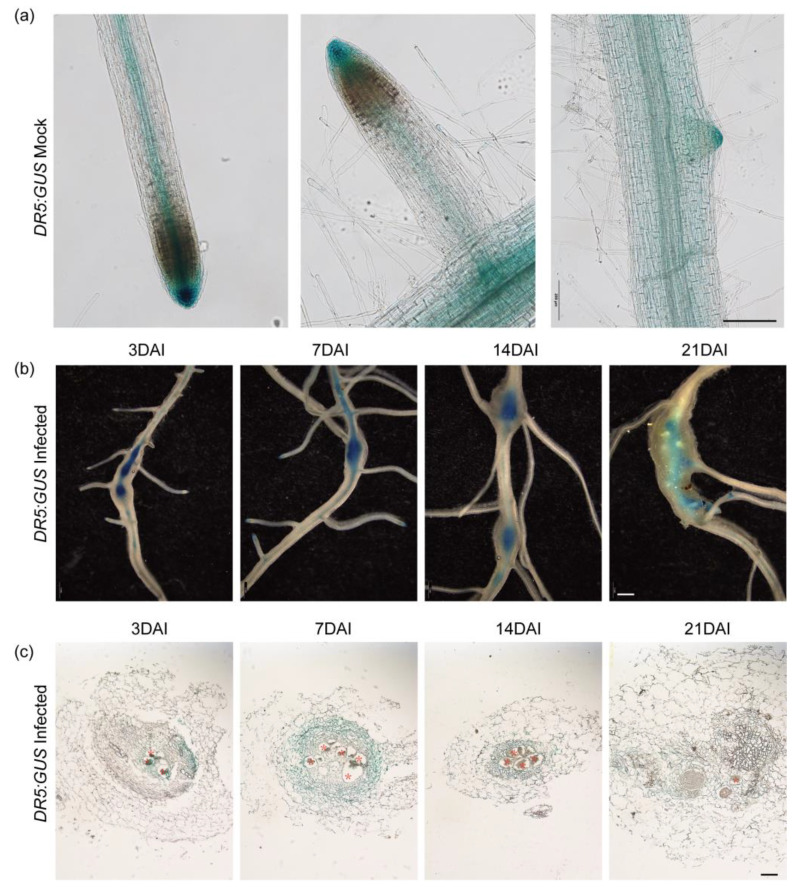
DR5 promoter activity analysis showed the auxin location in transgenic roots infected with *M. incognita*. (**a**) The GUS staining of *DR5:GUS* pattern in cucumber transgenic hairy roots with mock infection, bar: 200 μm. (**b**) The GUS staining of *DR5:GUS* pattern in cucumber transgenic hairy roots with *M. incognita* infection, DAI: Days after inoculation, Bar: 1 mm. (**c**) Paraffin sections of root galls showed the concentrated *GUS* staining surrounding the giant cells. The asterisk represents giant cells, DAI: Days after inoculation, bar: 100 μm.

Auxins are known to regulate lateral root development, and they are also closely related to the process of root gall formation after root-knot nematode infection. Based on the transgenic hairy roots, the *DR5:GUS* reporter system was used to monitor auxin response and distribution in cucumber roots and root galls during nematode parasitism (Figure 3). In the mock-infected cucumber roots, the induction of the auxin response in root tips and lateral root primordia was also detected (Figure 3a). The vascular bundles also showed characteristic GUS staining (Figure 3a).

To determine the auxin response during nematode parasitism, the transgenic hairy roots were infected with nematodes. The results showed *DR5:GUS* auxin response is increased in root galls compared with the non-infected area (Figure 3b). At all the sampled stages, the GUS staining showed the same trends, which demonstrate that the activation of auxin signaling occurred specifically in infected root galls. Furthermore, root cross-sections of GUS-stained *DR5:GUS* transgenic hairy roots allowed us to localize the GUS signal to root cortex cells and giant cells in the vascular bundle (Figure 3c). Especially at the early stages (3 and 7 DAI) after infection with nematodes, the giant cells showed darker GUS staining compared with the GCs at 14 and 21 DAI (Figure 3c). The surrounding distribution of GUS signals showed the potential role in galls induced by nematode infection.

### 3.3. Nematode Infection Activated Cucumber Auxin Transport Genes

**Figure 4:**
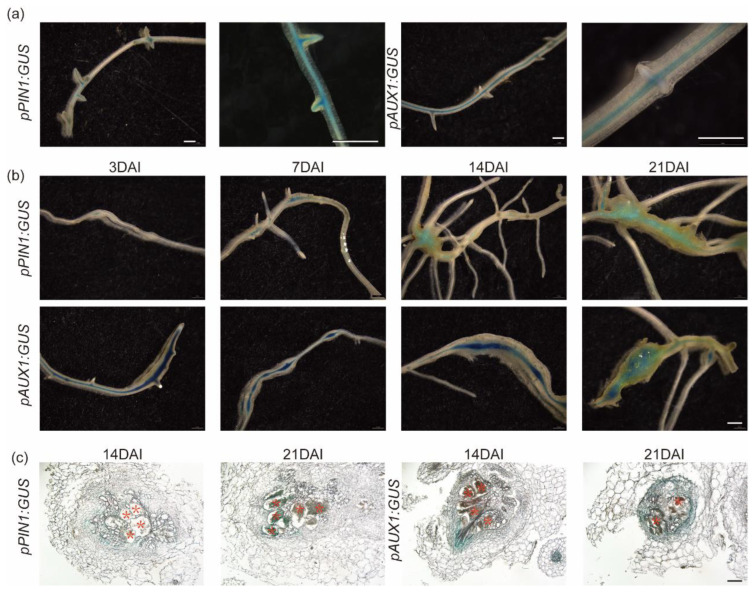
The promoter activity of *CsPIN1* and *CsAUX1* was activated during nematode parasitism. (**a**) The GUS staining of *pCsPIN1:GUS* and *pCsAUX1:GUS* pattern in cucumber transgenic hairy roots with mock infection. Bar: 1 mm. (**b**) Transgenic hairy roots harboring *pCsPIN1:GUS* and *pCsAUX1:GUS* expression pattern during *M. incognita* infection at 3, 7, 14, and 21 DAI. DAI: Days after inoculation. Bar: 1 mm. (**c**) Paraffin sections of root galls in transgenic hairy roots harboring *pCsPIN1:GUS* and *pCsAUX1:GUS* showed the concentrated GUS staining. The asterisk represents giant cells. Bar: 100 μm.

Pin-formed (PIN) and auxin-resistant (AUX) transporters are important for polar auxin transport, organogenesis, and long-distance auxin transport. We generated *pPIN1:GUS* and *pAUX1:GUS* reporter systems to monitor the expression of *CsPIN1* and *CsAUX1* during nematode parasitism (Figure 4). In the mock-infected transgenic roots, the GUS staining showed that these two genes were both expressed in the vascular bundles, but *CsPIN1* characteristically localized in the root vascular surrounding the lateral roots (Figure 4a). *CsPIN1* showed strong GUS staining in the lateral root primordia, especially the lateral root junctions, whereas *CsAUX1* was not detected in GUS activity (Figure 4a). During nematode parasitism, GUS staining showed that promoters of both genes shared similar expression patterns in the cucumber root galls (Figure 4b). At 14 and 21 DAI, the transgenic hairy roots transformed with *pPIN1:GUS* decreased compared with the early stages. However, *pAUX1:GUS* showed strong GUS staining signals at all developmental stages (Figure 4b). The results demonstrated the manipulation of auxin transport in cucumber roots during nematode parasitism.

The galls developed in transgenic roots sampled at 14 and 21 DAI were further processed by paraffin embedding technique to obtain transverse paraffin sections (Figure 4c). The results showed that both *CsPIN1* and *CsAUX1* were expressed in the cortex cells that surround the giant cells, which are involved in polar auxin transport. Unlike *CsAUX1*, the transgenic root transformed with *pPIN1:GUS* pattern also showed GUS signals in giant cells (Figure 4c).

**Figure 5:**
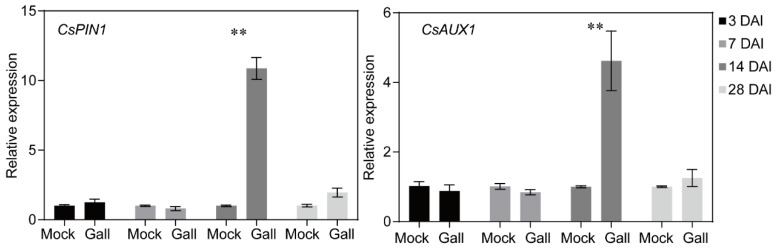
Expression of *CsPIN1* and *CsAUX1* was activated during *M. incognita* parasitism. Relative expression of *CsPIN1* and *CsAUX1* at different infected periods at transcript levels. Mock: Cucumber roots with mock inoculation; Galls: Cucumber root galls. *p*-values calculated using Student’s *t*-test, n = 4. ** *p* < 0.01.

We also detected the mRNA expression levels of *CsPIN1* and *CsAUX1* in different parts of cucumber roots by real-time fluorescence quantitative PCR (Figure 5). Nematode infection at 14 DAI produced rapid changes in the relative expression of both genes; the expression of *CsPIN1* and *CsAUX1* in root galls and the parts with galls removed increased significantly (*p* < 0.05). In contrast, we observed a significant downregulation of *CsPIN1* and *CsAUX1* in nematode-infected roots at 7 DAI (Figure 5). The other infection stages did not show significant changes.

### 3.4. TIBA Treatment Affects Cucumber Root Growth and Nematode Infection

**Figure 6:**
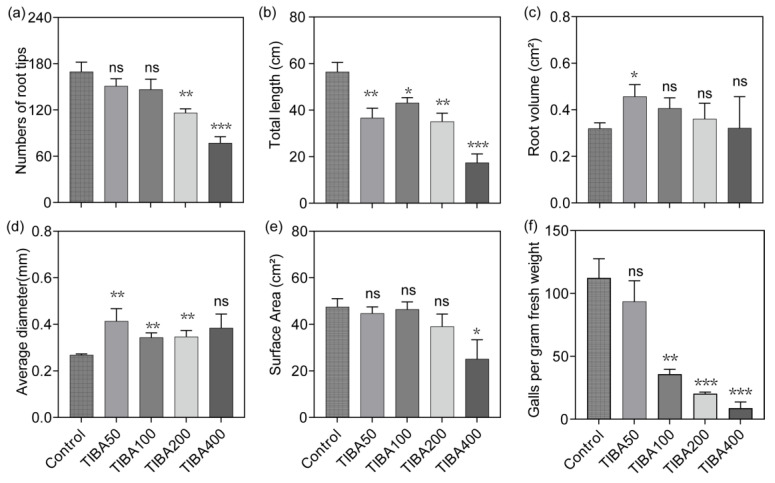
TIBA treatment affects cucumber root architecture and *M. incognita* infection. (**a**) The numbers of cucumber root tips treated with Mock and *M. incognita*. (**b**) The total length of cucumber root treated with Mock and *M. incognita*. (**c**) The root volume of cucumber treated with Mock and *M. incognita*. (**d**) The average diameter of cucumber root system treated with Mock and *M. incognita*. (**e**) The surface area of cucumber root system treated with Mock and *M. incognita*. (**f**) The status of *M. incognita* infection was evaluated using the numbers of root galls by the fresh root weight. *p*-values calculated using Student’s *t*-test, n = 8. * *p* < 0.05, ** *p* < 0.01, *** *p* < 0.001; ns, not significant.

To explore whether auxin transport leads to nematode parasitism, 2,3,5-triiodobenzoic acid (TIBA), a well-known auxin transport inhibitor [24], was applied to cucumber roots infected with *M. incognita*. The number of tips and total length of cucumber roots decreased with gradient TIBA concentrations as expected (Figure 6a,b). The highest concentration (400 μM) significantly decreased the surface area of cucumber roots (*p* < 0.05), but other concentrations did not show significant changes (Figure 6e). The average diameter of cucumber roots under *M. incognita* parasitism increased, but the root volume did not show obvious changes due to the decreased total length (Figure 6c,d). The galls developed in cucumber roots were further counted to evaluate the effect of TIBA treatment on nematode infection. The results showed TIBA treatments can reduce the numbers of cucumber root galls. In the TIBA treatment, 200 and 400 μM showed the most pronounced inhibitory effect (Figure 6f) which showed possible involvement of auxin transport in *M. incognita* parasitism of cucumber.

## 4. Discussion

The data reported here on cucumber root architecture after nematode infection validate previous studies showing that nematode parasitism can affect the morphology of plant root systems [25]. Because *Meloidogyne incognita* completes its life cycle inside the host root and feeds on living tissue, the effects on the host root translate into the formation of galls, which could destroy its diverse functions [26,27]. The significant decrease in root tips showed the inhibition of cucumber lateral root development during nematode parasitism (Figure 1h). However, the abnormal growth of lateral roots on the cucumber root galls indicated that the process of root development is affected during the development of nematodes in the root (Figure 2). This demonstrated that nematodes can induce new lateral root formation in galls of cucumber roots by using different molecular pathways involved in root development. It has been observed that root galls frequently contain LR primordia and induce the de novo organogenesis of LRs with no canonical left-right alternate pattern in Arabidopsis during *M. javanica* parasitism [20]. To absorb more nutrients, *Meloidogyne incognita* infection affected the root morphology of peanut, especially increasing the lateral root number [28] which may be associated with the growth of cucumber lateral roots in galls.

The process of gall and GC differentiation in root vascular cells undergoes massive molecular changes [27,29,30] to lead to the formation of highly specialized feeding sites. The vital role of the plant hormone auxin in lateral root development has been proven [31] but the mechanism of auxin involvement in nematode infection remains incomplete. In short, both lateral root development and nematode feeding site formation are controlled by the plant hormone auxin [13,32].

In this study, we showed the auxin response during nematode parasitism, including the early invading stage and the later development stages (Figure 3), which indicated functions of auxin in cucumber roots under nematode stress. We used the synthetic auxin-inducible promoter DR5, which is the most widely used sensor to monitor auxin response and distribution [33]. Reporters show that the cells surrounding giant cells display an auxin response as early as 1 week after infection [6]. The paraffin sections of galls developed in *DR5:GUS* transgenic cucumber hairy roots also showed the same results (Figure 3c). The functions of auxin depended on the transport process, which can lead to the accumulation of auxins at specific locations. In plant roots, the transmembrane proteins of the AUX1 act as influx carriers of auxin, and efflux occurs through the PIN-FORMED (PIN) protein family [6,16,34]. Therefore, we further analyzed the promoter activity of *CsPIN1* and *CsAUX1* to evaluate the effects of nematode parasitism on auxin transport (Figure 3). The GUS staining of transgenic hairy roots transformed with *CsPIN1* and *CsAUX1* promoter GUS vector showed the same results as *DR5:GUS* (Figure 4), which demonstrated the accumulation of auxin in root galls. Our results indicated the expression of *CsPIN1* in cucumber root galls and cells surrounding the GCs (Figure 4b,c), which is consist with previous studies [13]. In syncytia induced by CNs, *PIN1* expression is downregulated in young syncytia, and pin1 mutants lead to significantly fewer and smaller cysts [35]. Previously, the CsPIN1 cDNA encoding a PIN auxin efflux facilitator was identified in cucumber [36]. In this study, the repression of *CsPIN1* was decreasing at 7 DAI but significantly increased at 14 DAI (Figure 5). This indicated different regulation of auxin homeostasis during different nematode developmental stages. The PIN1 proteins are localized at the plasma membrane and facilitate cellular auxin efflux [37]; as a result, the GUS staining showed promotion at early stages (Figure 4). The increased expression of the AUX1 importer was detected in young feeding sites during CN infection process potentially resulting in a greater auxin influx [38]. Auxin influx transporter (AUX1) is suggested to have a role in the establishment and maintenance of nematode feeding sites by auxin transporting. The levels of auxin have been found to be increased in nematode-infected roots and are essential for syncytia formation [39,40,41]. Transcriptome analysis of galls also showed AUX1 was induced in giant cells [42]. In the cucumber transgenic hairy roots, *M. incognita* infection induced GUS signals of *CsAUX1* promoter, which indicated the continuous and strong influx of auxin into the root galls (Figure 4b). Previous research proposed that GC initiation and gall expansion depend on auxin import through AUX1 which affects nematode development [13]. Studies on cell expansion showed that GC development in host roots may require AUX1 [43].

The TIBA treatment, which perturbs auxin efflux, was used to explore the effects of auxin transport disruption on nematode parasitism. The inhibition of total length and root tips on cucumber roots showed the importance of auxin in root development (Figure 6). In rice seedlings, TIBA treatments decrease the root numbers compared with IAA treatments [44]. We further analyzed gall development by infection with *M. incognita* which indicated that TIBA treatment resulted in fewer galls in cucumber roots (Figure 6f). Several studies have demonstrated that auxin is essential to promote LR initiation [32,45], which is basically consistent with the results in this paper. Moreover, auxin is known to play a role in root elongation, which is the zone where RKNs infect host roots and begin their feeding sites [46]. This indicates that auxin is likely involved in the expansion of the GCs. *n* this process, the regulation of lateral roots on galls may be related to the coordinated control of nematode feeding site development by auxin.

## 5. Conclusions

In this study, the phenomenon of abnormal lateral root growth on cucumber root galls may be attributed to changes in auxin within the GCs induced by *M. incognita*. Using the described results, we suggest that auxin plays a vital role in nematode infection. Moreover, *CsPIN1* and *CsAUX1* are the driving forces for the accumulation of auxin in nematode feeding site development. During nematode parasitism, auxin may contribute to the inducement of lateral roots on cucumber root galls. Our results provide evidence for a redirected flow of endogenous auxin within the plant during RKN feeding site development and the involved lateral root development.

## Data Availability

Data are contained within the article.

## Supplementary Materials

The following supporting information can be downloaded at: https://www.mdpi.com/article/10.3390/plants13192679/s1, Table S1: Primers for GUS reporter constructs; Table S2: Primers for qPCR
