# Supplementary material for: Auxin-Mediated Lateral Root Development in Root Galls of Cucumber under Meloidogyne incognita Stress
Source: Plants (Basel). 2024 Sep 24;13(19):2679. doi: 10.3390/plants13192679 (PMC11478513; doi:10.3390/plants13192679)
Supplement: Supplementary file 1 [file plants-13-02679-s001.zip › plants-3191346-supplementary.pdf]

TableS1 Primers for GUS reporter constructs

Primers	Accession number	sequences
CsPIN1-1391-F	Csa1G042820	GCAGGTCGACGGATCCCCGGTCGGTGCATTAGTCACAAAGTT
CsPIN1-1391-R		CCATGGTGGACTCCTCTTAGTGGGGTTTGTGTTAGGCTTTG
CsAUX1-1391-F	Csa3G731880	GCAGGTCGACGGATCCCCGGCGTTTCTATCACTGTCTGTCAAT
CsAUX1-1391-R		CCATGGTGGACTCCTCTTAGTTGTTCTGATCGATAAGGTCTC

TableS2 Primers for qPCR

Primers	sequences
qCsPIN1-F	TCTCCTGGAAAAGTAGAAGGACG
qCsPIN1-R	TAGTGGGTGGCATGGTTTTGG
qCsAUX1-F	GGGCTTTCGGAGATGAGCTT
qCsAUX1-R	CCCACACAAAGTACAACGGC
qCsTUA-F	CATTCTCTCTTGGAACACACTGA
qCsTUA-R	TCAAACTGGCAGTTAAAGATGAAA
